# A Viscidane Diterpene and Polyacetylenes from Cultures of *Hypsizygus marmoreus*

**DOI:** 10.1007/s13659-015-0058-2

**Published:** 2015-03-28

**Authors:** Ling Zhang, Zheng-Hui Li, Ze-Jun Dong, Yan Li, Ji-Kai Liu

**Affiliations:** State Key Laboratory of Phytochemistry and Plant Resources in West China, Kunming Institute of Botany, Chinese Academy of Sciences, Kunming, 650204 China

**Keywords:** *Hypsizygus marmoreus*, Viscidane diterpene, Polyacetylenes

## Abstract

**Abstract:**

Investigation on the cultures of *Hypsizygus marmoreus* resulted in the isolation of a new viscidane diterpene, 8-oxoviscida-3,11(18)-diene-13,14,15,19-tetraol (**1**) and two new polyacetylenes, (*E*)-10-(1,1-dimethyl-2-propenyloxy)-2-decene-4,6,8-triyn-1-ol (**2**) and 10-(1,1-dimethyl-2-propenyloxy)deca-4,6,8-triyn-1-ol (**3**), together with two known polyacetylenes, (*E*)-2-decen-4,6,8-triyn-1-ol (**4**) and 4,6,8-decatriyn-1-ol (**5**). Their structures were elucidated on the basis of extensive spectroscopic studies. Compound **1** is the first finding of viscidane diterpene in mushrooms. Compounds **1**, **3** and **5** were tested for cytotoxicity against human tumor cell lines HL-60, SMMC-7721, A-549, MCF-7 and SW-480. None of the compounds showed cytotoxic activity (IC_50_ > 40 µM).

**Graphical Abstract:**

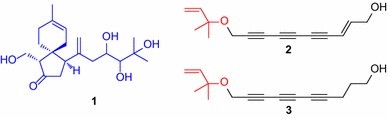

**Electronic supplementary material:**

The online version of this article (doi:10.1007/s13659-015-0058-2) contains supplementary material, which is available to authorized users.

## Introduction

The fungus *Hypsizygus marmoreus* (Tricholomataceae) is a group of edible mushrooms native to East Asia, occurring in autumn and winter of the north temperature zone. In a previous investigation of their fruiting bodies led to the isolation of steroids [1, 2], sphingolipids [3], proteins [4], and polyisoprenepolyols [5–7]. However, the cultures of this mushroom have not been chemically investigated so far. In continuation of our study of the secondary metabolites from the untapped resources of higher fungi collected in China [8–11], we have investigated the cultures of *H. marmoreus*, which led to the isolation of three new compounds, a viscidane diterpene (**1**) and two polyacetylenes (**2** and **3**), as well as two known polyacetylenes (**4** and **5**). Viscidane diterpenes are characteristic constituents in *Eremophila* species, nevertheless, **1** is the first example of this type found in higher fungi. Furthermore, polyacetylenes usually possess high antifungal and nematicidal activities and are phototoxic against certain viruses [12–16]. In macrofungi, the incorporation of oleic, linoleic, crepenynic, and dehydrocrepenynate into polyacetylenes is well established [17]. The present paper reports the isolation and structure determination of the new compounds.

## Results and Discussion

An EtOAc extracts (4.0 g) of the culture broth (18 L) of *H. marmoreus* was subjected to silica gel column chromatography (CC) with a gradient elution system of petroleum ether–acetone (100:0–0:100) to obtain eight fractions. Fractions 3, 5 and 7 were further chromatographed on Sephadex LH-20 CC (CHCl_3_–MeOH, 1:1) and purified by preparative HPLC (Pre-HPLC, MeCN–H_2_O) to give three new compounds **1**–**3** and known ones. The known compounds were determined to be (*E*)-2-decen-4,6,8-triyn-1-ol (**4**) [18] and 4,6,8-decatriyn-1-ol (**5**) [19].

**Fig. 1:**
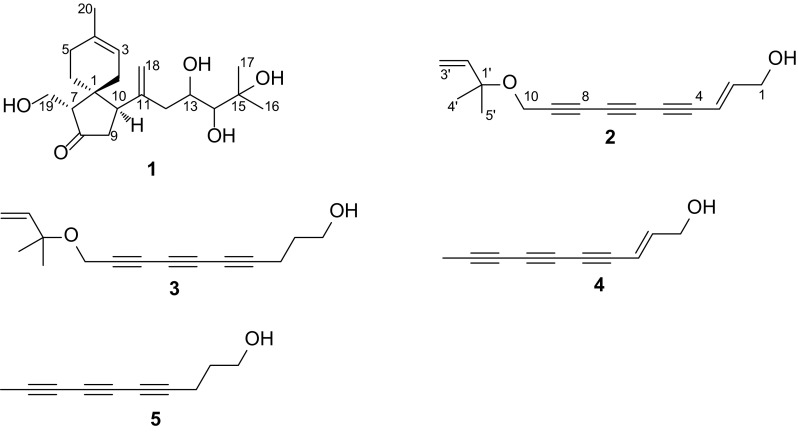
Structures of compounds **1–5**

**Fig. 2:**
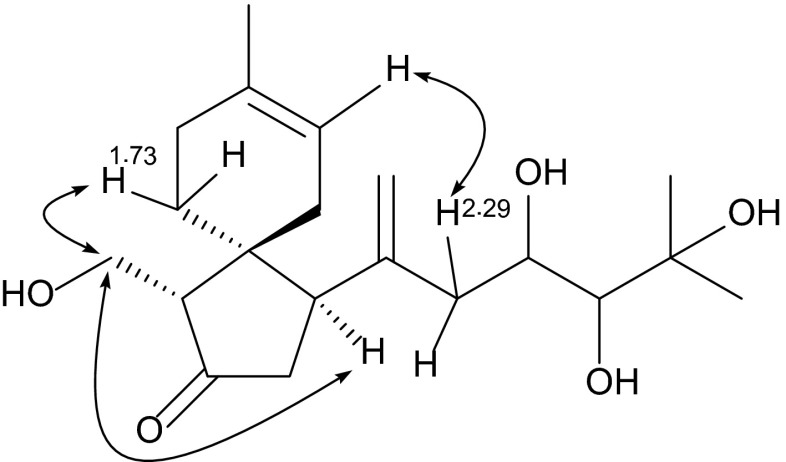
Key ROESY correlations of **1**

Compound **1**, obtained as oil, had the molecular formula C_20_H_32_O_5_ based on the HRESIMS (pos.), showing a quasi-molecular ion peak at *m*/*z* 375.2147 (calcd for C_20_H_32_O_5_Na, 375.2147) with five degrees of unsaturation. In accordance with the molecular formula, 20 carbon resonances were resolved in the ^13^C NMR spectrum (Table 1), including a saturated ketone at *δ*_C_ 222.0 (s), a set of signals at *δ*_C_ 149.0 (s), 134.3 (s), 121.4 (d), 114.6 (t) assignable to a terminal double bond and a trisubstituted one, three oxygen-bearing carbons at *δ*_C_ 78.7 (d), 74.6 (s) and 70.2 (d), as well as three methyl signals at *δ*_C_ 27.2 (q), 26.4 (q) and 23.5 (q). The ^1^H NMR spectrum (Table 1), in combination with the HSQC spectrum, exhibited three vinyl signals at *δ*_H_ 5.35 (1H, br. s), 5.08 (1H, br. s) and 4.77 (1H, br. s), four oxygen-bearing protons at *δ*_H_ 4.01 (1H, br. dd, *J* = 8.7, 4.7 Hz), 3.83 (1H, dd, *J* = 11.4, 5.3 Hz), 3.71 (1H, dd, *J* = 11.4, 5.8 Hz) and 3.06 (1H, br. s), three spin-coupled protons at *δ*_H_ 3.05 (1H, dd, *J* = 8.8, 4.4 Hz), 2.53 (1H, dd, *J* = 19.4, 8.8 Hz) and 2.41 (1H, dd, *J* = 19.4, 4.4 Hz), and three tertiary methyls signals at *δ*_H_ 1.67 (3H, s), 1.24 (3H, s), and 1.22 (3H, s). According to the degrees of unsaturation, this molecule contained two rings. The above NMR character was very similar with that of a known compound viscida-3,11(18),14-triene [20]. Nevertheless there were obvious differences: the absence of a trisubstituted double bond signals, a doublet methyl signals and two high field methylene signals, instead a saturated carbonyl carbon signal and four oxygen-bearing carbon signals were observed. The carbonyl and hydroxymethyl were posited at C-8 and C-7 respectively, as established by the HMBC correlations (Table 1) of *δ*_H_ 3.83 (1H, dd, *J* = 11.4, 5.3 Hz, H-19a) and 3.71 (1H, dd, *J* = 11.4, 5.8 Hz, H-19b) with *δ*_C_ 45.1 (s, C-1), 58.7 (d, C-7) and 222.0 (s, C-8); of *δ*_H_ 2.45 (1H, dd, *J* = 5.8, 5.3 Hz, H-7), 2.53 (1H, dd, *J* = 19.4, 8.8 Hz, H-9a), 2.41 (1H, m, H-9b), 3.05 (1H, m, H-10), 3.83 (1H, dd, *J* = 11.4, 5.3 Hz, H-19a) and 3.71 (1H, dd, *J* = 11.4, 5.8 Hz, H-19b) with *δ*_C_ 222.0 (s, C-8). HMBC correlations also evidenced that C-13, C-14 and C-15 in **1** were hydroxylated. From ROESY experiment (Fig. 2), the significant correlations of H-10/H-19 and H-19/H-6 were observed, indicating *α*-orientation of these protons. In combination with the comparison of relevant NMR chemical shifts and ^1^H–^1^H coupling constants with those of analogues [21, 22], the configuration of **1** was established as shown in Fig. 1. Accordingly, the structure of **1** was determined and named as 8-oxoviscida-3,11(18)-diene-13,14,15,19-tetraol.

**Table 1:** NMR spectroscopic data and HMBC correlations for **1** in CD_3_OD

No.	*δ*_H_	*δ*_C_	HMBC (H → C)
1		45.1 (s)	
2	2.13 (1H, br. d, 17.9); 2.03 (1H, br. d, 17.9)	33.1 (t)	C-1, C-3, C-4, C-6
3	5.35 (1H, br. s)	121.4 (d)	C-1, C-5, C-20
4		134.3 (s)	
5	2.18 (1H, m); 2.04 (1H, m)	28.6 (t)	C-1, C-3, C-4, C-6
6	1.73 (1H, m); 1.59 (1H, m)	28.8 (t)	C-1, C-2, C-4, C-5, C-7
7	2.45 (1H, dd, 5.8, 5.3)	58.7 (d)	C-2, C-6, C-8, C-9, C-19
8		222.0 (s)	
9	2.53 (1H, dd, 19.4, 8.8); 2.41 (1H, dd, 19.4, 4.4)	43.6 (t)	C-7, C-8, C-10, C-11
10	3.05 (1H, dd, 8.8, 4.4)	44.3 (d)	C-1, C-6, C-7, C-8, C-11, C-18
11		149.0 (s)	
12	2.38 (1H, dd, 14.0, 8.7); 2.29 (1H, dd, 14.0, 4.7)	44.9 (t)	C-10, C-11, C-13, C-14, C-18
13	4.01 (1H, br.dd, 8.7, 4.7)	70.2 (d)	C-11, C-12, C-15
14	3.06 (1H, br. s)	78.7 (d)	C-12, C-15, C-17
15		74.6 (s)	
16	1.24 (3H, s)	27.2 (q)	C-14, C-15, C-17
17	1.22 (3H, s)	26.4 (q)	C-14, C-15, C-16
18	5.08 (1H, br. s); 4.77 (1H, br. s)	114.6 (t)	C-10, C-11, C-12
19	3.83 (1H, dd, 11.4, 5.3); 3.71 (1H, dd, 11.4, 5.8)	59.5 (t)	C-1, C-7, C-8
20	1.67 (3H, s)	23.5 (q)	C-3, C-4, C-5

Compound **2** was isolated as yellow oil. HREIMS analysis gave the molecular formula C_15_H_16_O_2_ (*m*/*z* 228.1158, calcd 228.1150), requiring eight degrees of unsaturation. The compound showed a UV spectrum typical of a triyne at 331, 309, 291, 274, 258 and 244 nm [19], whereas its IR spectrum indicated the presence of hydroxy (3406 cm^−1^), triyn (2179 cm^−1^) and acetylene (1626, 946 cm^−1^). The ^1^H NMR spectrum (Table 1) showed resonances for a terminal double bond at *δ*_H_ 5.80 (1H, dd, *J* = 17.4, 11.1 Hz, H-2′), 5.18 (1H, br. d, *J* = 11.1 Hz, H-3′) and 5.17 (1H, br. d, *J* = 17.4 Hz, H-3′), a *trans* disubstituted double bond at *δ*_H_ 6.48 (1H, dt, *J* = 15.9, 4.5 Hz, H-2) and 5.83 (1H, d, *J* = 15.9 Hz, H-3), two oxygenated methylene protons at *δ*_H_ 4.27 (2H, d, *J* = 4.5 Hz, H-1) and 4.09 (2H, s, H-10), and two methyl signals at *δ*_H_ 1.31 (6H, s, H-4′ and H-5′). In combination with HMQC spectrum, 15 protons were unambiguously assigned to their corresponding carbon atoms. However, according to the molecular formula, one proton signal was not observed, which can presumably be attributed to a hydroxy group. The ^13^C NMR (DEPT) spectrum (Table 2) exhibited 15 carbon signals, consisting of two methyls, three methylenes (including two oxidized carbons), three olefinic methines, and seven quaternary carbons (including a triyn group). The ^1^H and ^13^C NMR data of **2** were similar to those of (*E*)-2-decen-4,6,8-triyn-1-ol (**4**) [18], except for the additional signals at *δ*_C_ 142.5 (d, C-2′), 115.2 (t, C-3′), 77.1 (s, C-1′), 25.7 (q, C-4′ and C-5′) and 51.9 (t, C-10), and the absence of the methyl signal at *δ*_C_ 4.6 (q, C-10), indicating that the methyl at C-10 in **4** was replaced by a reverse isoprene 2-methylbut-3-en-2-yloxy moiety in **2**. This conclusion was supported by the HMBC correlations from 5.18 (1H, br. d, *J* = 11.1 Hz, H-3′) and 5.17 (1H, br. d, *J* = 17.4 Hz, H-3′) to 142.5 (d, C-2′), 77.1 (s, C-1′) and 25.7 (q, C-4′ and C-5′), from 4.09 (2H, s, H-10) to 77.1 (s, C-1′), and from 1.31 (6H, s, H-4′ and H-5′) to 142.5 (d, C-2′) and 77.1 (s, C-1′). The C-2/C-3 olefin was assigned the *E*-geometry based on the characteristic vicinal coupling constant of H-2 and H-3 (*J* = 15.9 Hz). Thus, the structure of **2** was determined as (*E*)-10-(1,1-dimethyl-2-propenyloxy)-2-decene-4,6,8-triyn-1-ol.

**Table 2:** NMR spectroscopic data of compounds **2** and **3** in CDCl_3_

No.	**2**		**3**	
	*δ*_H_	*δ*_C_	*δ*_H_	*δ*_C_
1	4.27 (d, 4.5)	62.6 t	3.73 (t, 6.3)	61.2 t
2	6.48 (dt, 15.9, 4.5)	147.3 d	1.78 (m)	30.6 t
3	5.83 (d, 15.9)	108.1 d	2.43 (t, 7.0)	15.9 t
4^a^		62.6 s		79.9 s
5^a^		75.2 s		65.9 s
6^a^		74.9 s		63.2 s
7^a^		66.1 s		75.0 s
8^a^		78.1 s		59.6 s
9^a^		70.1 s		70.1 s
10	4.09 (s)	51.9 t	4.05 (s)	51.8 t
1′		77.1 s		77.0 s
2′	5.80 (dd, 17.4, 11.1)	142.5 d	5.79 (dd, 17.2, 11.2)	142.4 d
3′	5.18 (br. d, 11.1) 5.17 (br. d, 17.4)	115.2 t	5.17 (br. d, 11.2) 5.16 (br. d, 17.2)	115.2 t
4′	1.31 (s)	25.7 q	1.29 (s)	25.7 q
5′	1.31 (s)	25.7 q	1.29 (s)	25.7 q

^a^These data may be interchanged

Compound **3** was also obtained as yellow oil. Its molecular formula was determined as C_15_H_18_O_2_ from the HREIMS ion peak at 230.1294 (C_15_H_18_O_2_, calcd 230.1307), representing an unsaturation value of seven. Comparing the NMR data of **3** (Table 2) with those of **2**, **3** showed evidence of having two more methylenes (*δ*_C_ 30.6 and 15.9) and two less olefinic methines (*δ*_C_ 147.3 and 108.1) than **1**. Further evidence from the HMBC spectrum of **3**, in which the proton at 2.43 (2H, t, *J* = 7.0 Hz, H-3) correlated to C-1 (61.2, t) and 30.6 (t, C-2), and the proton at 1.78 (2H, *q*-like, H-2) correlated to C-4 (79.9, s), C-1 (61.2, t), C-2 (30.6, t) and C-3 (15.9, t), suggesting that a double bond between C-2 and C-3 was hydrogenated in **3**. Therefore, compound **3** was assigned as 10-(1,1-dimethyl-2-propenyloxy)deca-4,6,8-triyn-1-ol.

## Experimental

### General Experimental Procedures

Optical rotations were measured on a Jasco P-1020 (Jasco International Co., Ltd., Tokyo, Japan) automatic digital polarimeter. IR spectra were recorded using a Bruker Tensor 27 FT-IR (Bruker Optics GmbH, Ettlingen, Germany) spectrometer with KBr pellets. UV spectra were carried out on a Shimadzu UV-2401A spectrometer. NMR spectra were carried out on Bruker DRX-500, AV-400 or AV-600 (Bruker BioSpin GmbH, Rheinstetten, Germany) spectrometer with the deuterated solvent as an internal standard. EIMS and ESIMS (including HRESIMS) were measured on Finnigan-MAT 90 and API QSTAR Pulsar i (MDS Sciex, Concord, Ontario, Canada) mass spectrometers, respectively. Silica gel 200–300 mesh (Qingdao Marine Chemical Inc., Qingdao, China) and Sephadex LH-20 (Amersham Biosciences, Uppsala, Sweden) were used for normal pressure column chromatography. Fractions were monitored and analyzed by TLC (silica gel 60 F_254_, Qingdao Marine Chemical Inc., Qingdao, China), in combination with Agilent 1200 series HPLC system (Eclipse XDB-C18 column, 5 μm, 4.6 × 150 mm). Preparative HPLC was performed using an Agilent 1100 series (Zorbax SB-C18 column, 5 μm, 9.4 × 150 mm).

### Fungal Material and Cultivation Conditions

The fungus *H. marmoreus* was provided by Huazhong Agricultural University (cultivated), in 2000, and identified by Prof. Yu-Cheng Dai, Beijing Forestry University. The voucher specimen (MC00110) was deposited at the State Key Laboratory of Phytochemistry and Plant Resources in West China, Kunming Institute of Botany, CAS. Culture PDA medium: potato (peeled), 200 g, glucose, 20 g, KH_2_PO_4_, 3 g, MgSO_4_, 1.5 g, citric acid, 0.1 g, and thiamin hydrochloride, 10 mg, in 1 L of deionized H_2_O. Reagent bottles were used as a flask (size: 500 mL; volume of media: 300 mL). The pH was adjusted to 6.5 before autoclaving, and the fermentation was carried out on a shaker at 25 and 150 rpm for 30 days.

### Extraction and Isolation

The culture broth (18 L) was extracted three times with EtOAc (36 L). The combined EtOAc extracts were evaporated in vacuo to give a residue (4.0 g). The residue was subjected to silica gel column chromatography (CC) with a gradient elution system of petroleum ether–acetone (100:0–0:100) to obtain eight fractions. Fraction 3 was eluted with petroleum ether–acetone (10:1). It was then subjected to Sephadex LH-20 CC (CHCl_3_–MeOH, 1:1) and Pre-HPLC (35–50 % MeCN in H_2_O over 40 min, 10 mL/min) to yield **2** (4.2 mg), **3** (12.5 mg), **4** (12.0 mg) and **5** (10.3 mg). The fraction 7 (petroleum ether–acetone, 3:1) was further chromatographed on Sephadex LH-20 CC (CHCl_3_–MeOH, 1:1) and then purified by Pre-HPLC (4–30 % MeCN in H_2_O over 40 min, 10 mL/min) to afford **1** (2.4 mg).

### 8-Oxoviscida-3,11(18)-diene-13,14,15,19-tetraol (**1**)

Oil, [α]_D_^10^ –3.9 (*c* 0.16, CH_3_OH). ^1^H and ^13^C NMR: see Table 1. ESIMS (pos.): 375 [M + Na]^+^. HRESIMS (pos.): 375.2147 (C_20_H_32_O_5_Na, calcd 375.2147).

### (*E*)-10-(1,1-Dimethyl-2-propenyloxy)-2-decene-4,6,8-triyn-1-ol (**2**)

Yellow oil. UV *λ*_max_ (MeOH): 331, 309, 291, 274, 258, 244 nm; IR (KBr): 3406, 3087, 2977, 2926, 2852, 2179, 1626, 946, 935 cm^−1^. ^1^H and ^13^C NMR: see Table 2. EIMS: 228 [M]^+^. HREIMS: 228.1158 (C_15_H_16_O_2_, calcd 228.1150).

### 10-(1,1-Dimethyl-2-propenyloxy)deca-4,6,8-triyn-1-ol (**3**)

Yellow oil. UV *λ*_max_ (MeOH): 283, 266, 253, 213, 204 nm; IR (KBr): 3405, 3086, 2978, 2933, 2854, 2218, 1718, 1626, 1145, 1057, 930 cm^−1^. ^1^H and ^13^C NMR: see Table 2. EIMS: 230 [M]^+^. HREIMS: 230.1294 (C_15_H_18_O_2_, calcd 230.1307).

### Cytotoxicity Assay

The following human tumor cell lines were used: HL-60, SMMC-7721, A-549, MCF-7 and SW-480. All the cells were cultured in RPMI-1640 or Dulbecco’s modified Eagle’s medium (DMEM) (Hyclone, USA), supplemented with 10 % fetal bovine serum (Hyclone, USA) at 37 °C in a humidified atmosphere with 5 % CO_2_. Cell viability was assessed by conducting colorimetric measurements of the amount of insoluble formazan that formed in living cells based on the reduction of 3-(4,5-dimethylthiazol-2-yl)-2,5-diphenyltetrazolium bromide (MTT) (Sigma, USA) [23]. Briefly, 100 mL of adherent cells were seeded into each well of 96-well cell culture plates and allowed to adhere for 12 h before drug addition, while suspended cells were seeded just before drug addition, both with an initial density of 1 × 10^5^ cells/mL in 100 μL of medium. Each tumor cell line was exposed to the tested compound at various concentrations in triplicate for 48 h, with 10-hydroxy-campto-thecine (Sigma, USA) as positive control. After the incubation, MTT (100 mg) was added to each well, and the incubation continued for 4 h at 37 °C. The cells were lysed with 100 μL of 20 % SDS–50 % DMF after removal of 100 μL of medium. The optical density of the lysate was measured at 595 nm in a 96-well microtiter plate reader (Bio-Rad 680, USA). The IC_50_ value of each compound was calculated by the Reed and Muench method [24].

## Electronic supplementary material

Below is the link to the electronic supplementary material.

Supplementary material 1 (PDF 10751 kb)
