# Supplementary material for: A Viscidane Diterpene and Polyacetylenes from Cultures of Hypsizygus marmoreus
Source: Nat Prod Bioprospect. 2015 Mar 28;5(2):99–103. doi: 10.1007/s13659-015-0058-2 (PMC4402581; doi:10.1007/s13659-015-0058-2)

Electronic Supplementary Material

A viscidane diterpene and polyacetylenes from cultures of *Hypsizygus marmoreus*

Ling Zhang, Zheng-Hui Li, Ze-Jun Dong, Yan Li and Ji-Kai Liu*

State Key Laboratory of Phytochemistry and Plant Resources in West China, Kunming Institute of Botany, Chinese Academy of Sciences, Kunming 650201, Yunnan, China

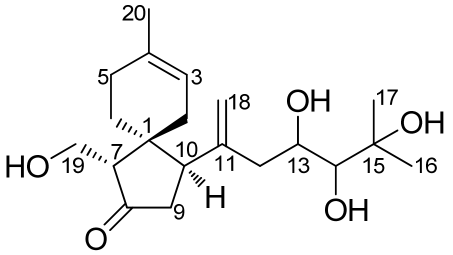

1

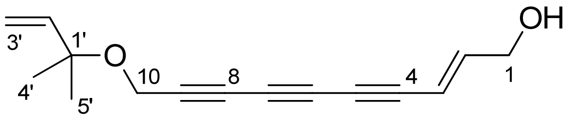

2

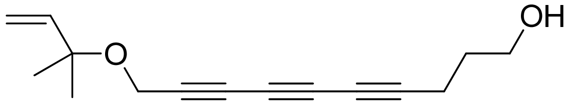

3

Structures of compounds **1**–**3**

*To whom correspondence should be addressed. E-mail: jkliu@mail.kib.ac.cn

Content list:

S1. ^{1}H NMR spectrum (CD_3OD) of 8-oxoviscida-3,11(18)-diene-13,14,15,19-tetraol (**1**).

S2. ^{13}C NMR (DEPT) spectrum (CD_3OD) of 8-oxoviscida-3,11(18)-diene-13,14,15,19-tetraol (**1**).

S3. HMBC spectrum (CD_3OD) of 8-oxoviscida-3,11(18)-diene-13,14,15,19-tetraol (**1**).

S4. HSQC spectrum (CD_3OD) of 8-oxoviscida-3,11(18)-diene-13,14,15,19-tetraol (**1**).

S5. COSY spectrum (CD_3OD) of 8-oxoviscida-3,11(18)-diene-13,14,15,19-tetraol (**1**).

S6. ROESY spectrum (CD_3OD) of 8-oxoviscida-3,11(18)-diene-13,14,15,19-tetraol (**1**).

S7. ^{1}H NMR spectrum ($CDCl_3$) of (*E*)-10-(1,1-dimethyl-2-propenyloxy)-2-decene-4,6,8-triyn-1-ol (**2**).

S8. ^{13}C NMR (DEPT) spectrum ($CDCl_3$) of (*E*)-10-(1,1-dimethyl-2-propenyloxy)-2-decene-4,6,8-triyn-1-ol (**2**).

S9. HMBC spectrum ($CDCl_3$) of (*E*)-10-(1,1-dimethyl-2-propenyloxy)-2-decene-4,6,8-triyn-1-ol (**2**).

S10. HSQC spectrum ($CDCl_3$) of (*E*)-10-(1,1-dimethyl-2-propenyloxy)-2-decene-4,6,8-triyn-1-ol (**2**).

S11. ^{1}H NMR spectrum ($CDCl_3$) of 10-(1,1-dimethyl-2-propenyloxy)deca-4,6,8-triyn-1-ol (**3**).

S12. ^{13}C NMR (DEPT) spectrum ($CDCl_3$) of 10-(1,1-dimethyl-2-propenyloxy)deca-4,6,8-triyn-1-ol (**3**).

S13. HMBC spectrum ($CDCl_3$) of 10-(1,1-dimethyl-2-propenyloxy)deca-4,6,8-triyn-1-ol (**3**).

S14. HSQC spectrum ($CDCl_3$) of 10-(1,1-dimethyl-2-propenyloxy)deca-4,6,8-triyn-1-ol (**3**).

S1. ^{1}H NMR spectrum (CD_3OD) of 8-oxoviscida-3,11(18)-diene-13,14,15,19-tetraol (**1**)

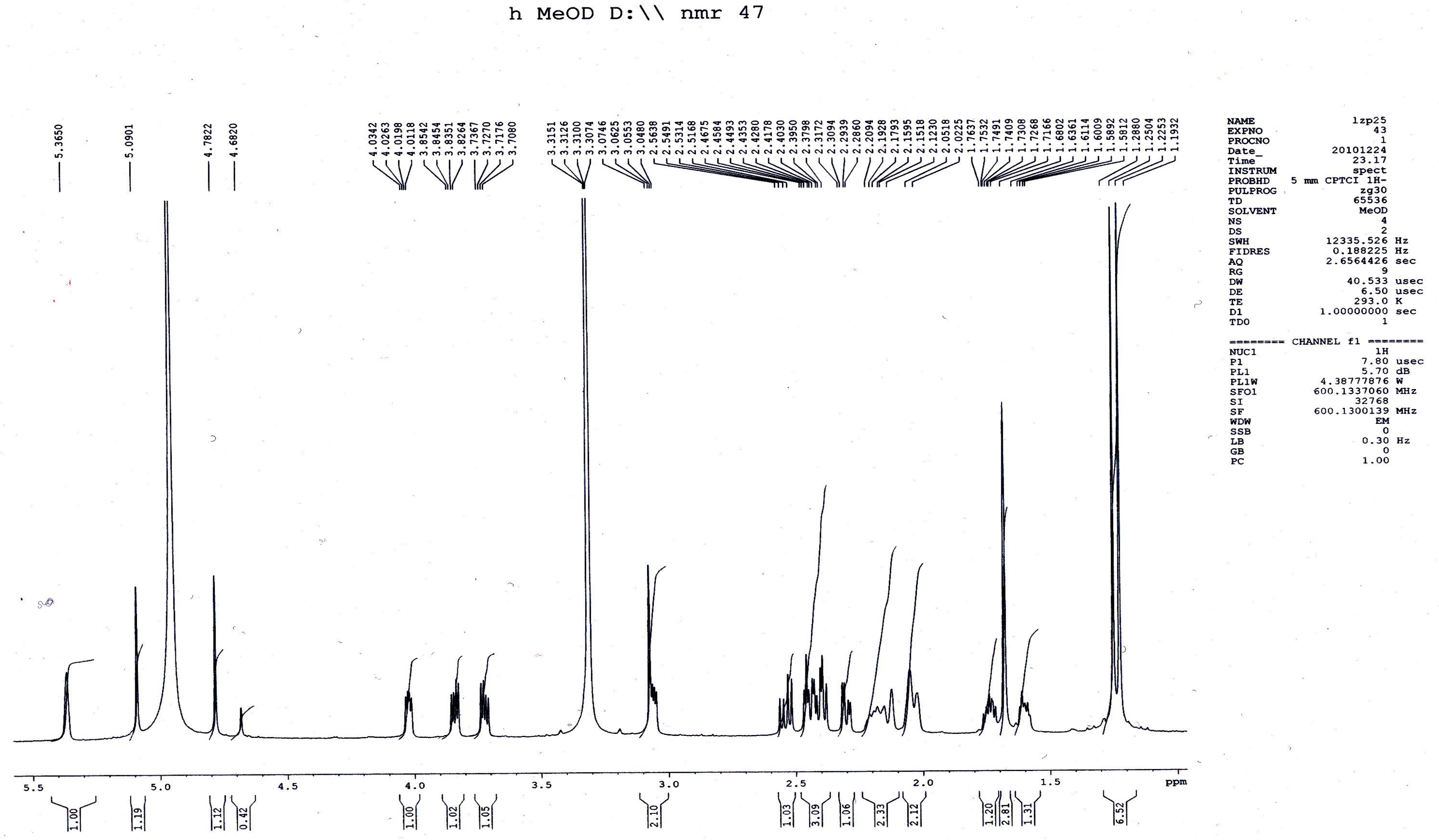

S2. ^{13}C NMR (DEPT) spectrum (CD_3OD) of 8-oxoviscida-3,11(18)-diene-13,14,15,19-tetraol (**1**)

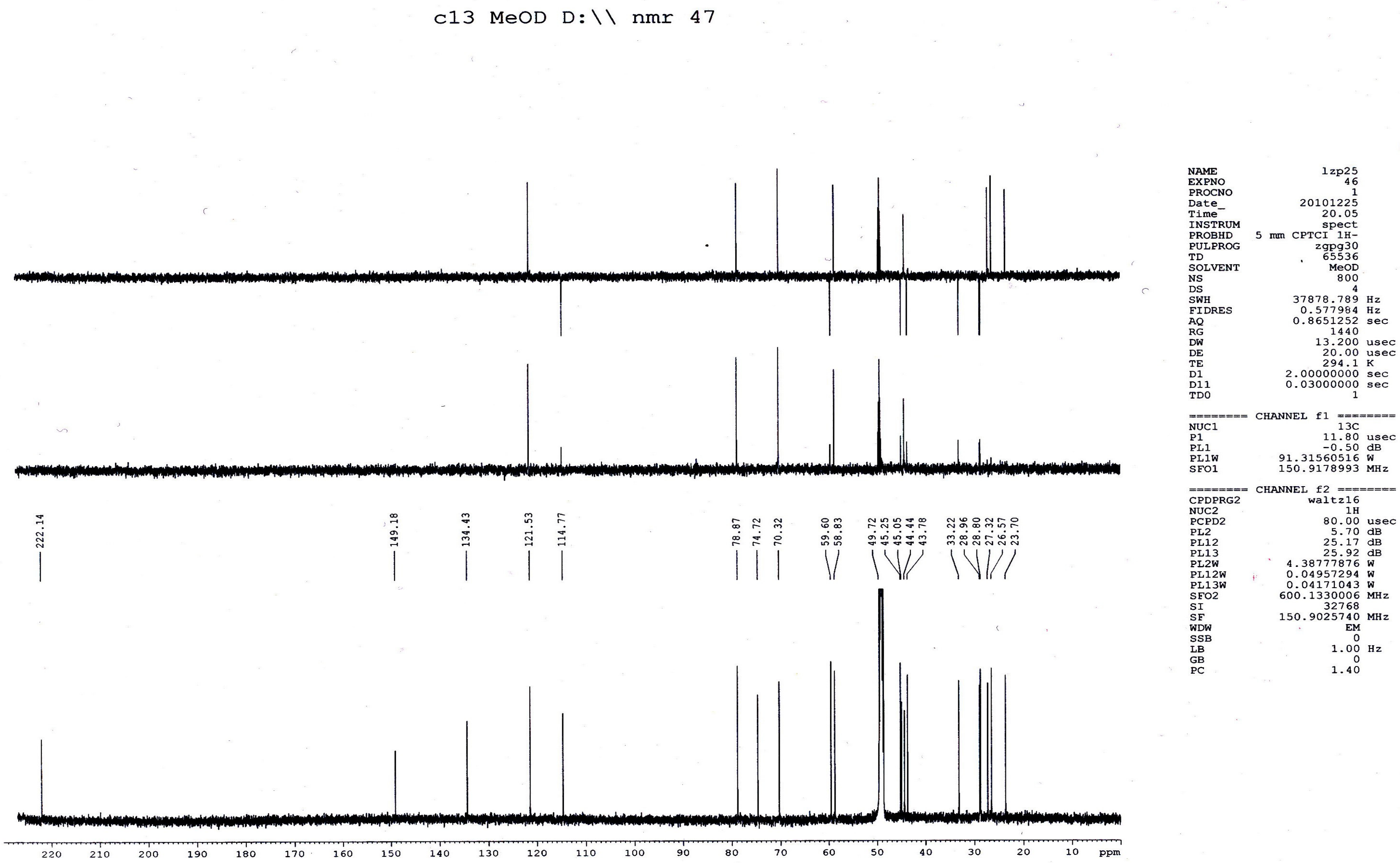

S3. HMBC spectrum (CD_3OD) of 8-oxoviscida-3,11(18)-diene-13,14,15,19-tetraol (**1**)

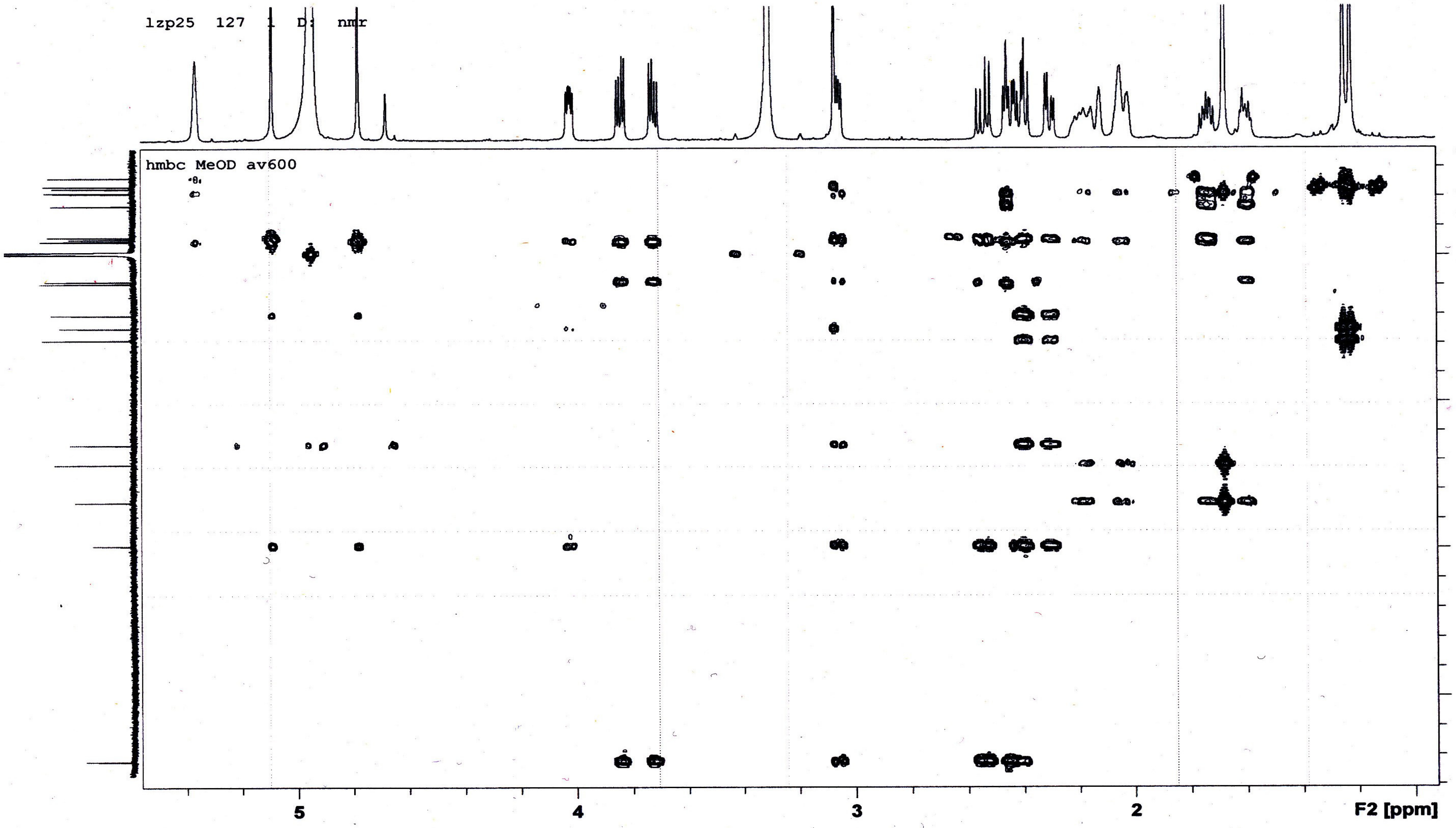

S4. HSQC spectrum (CD_3OD) of 8-oxoviscida-3,11(18)-diene-13,14,15,19-tetraol (**1**)

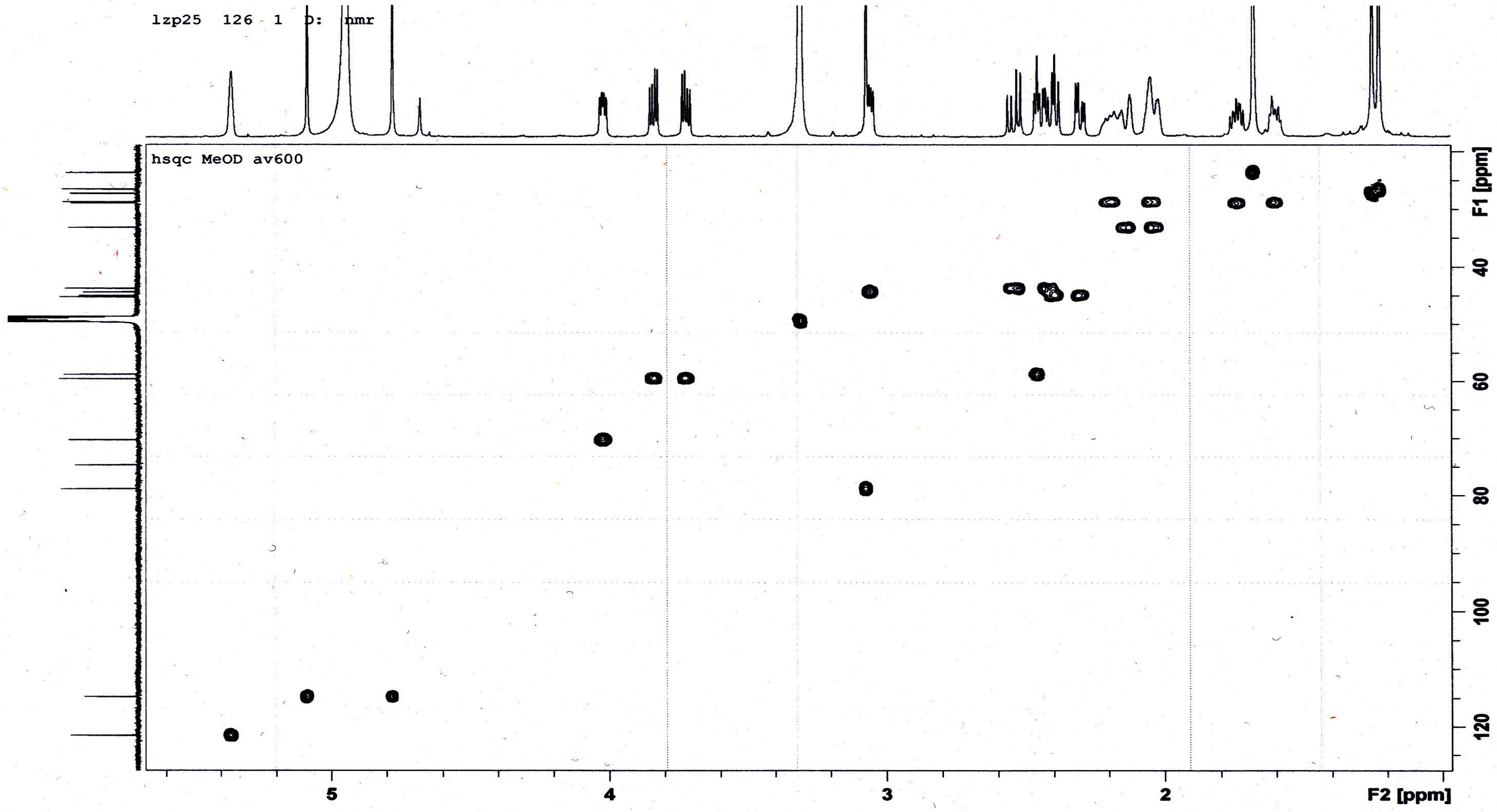

S5. COSY spectrum (CD_3OD) of 8-oxoviscida-3,11(18)-diene-13,14,15,19-tetraol (**1**)

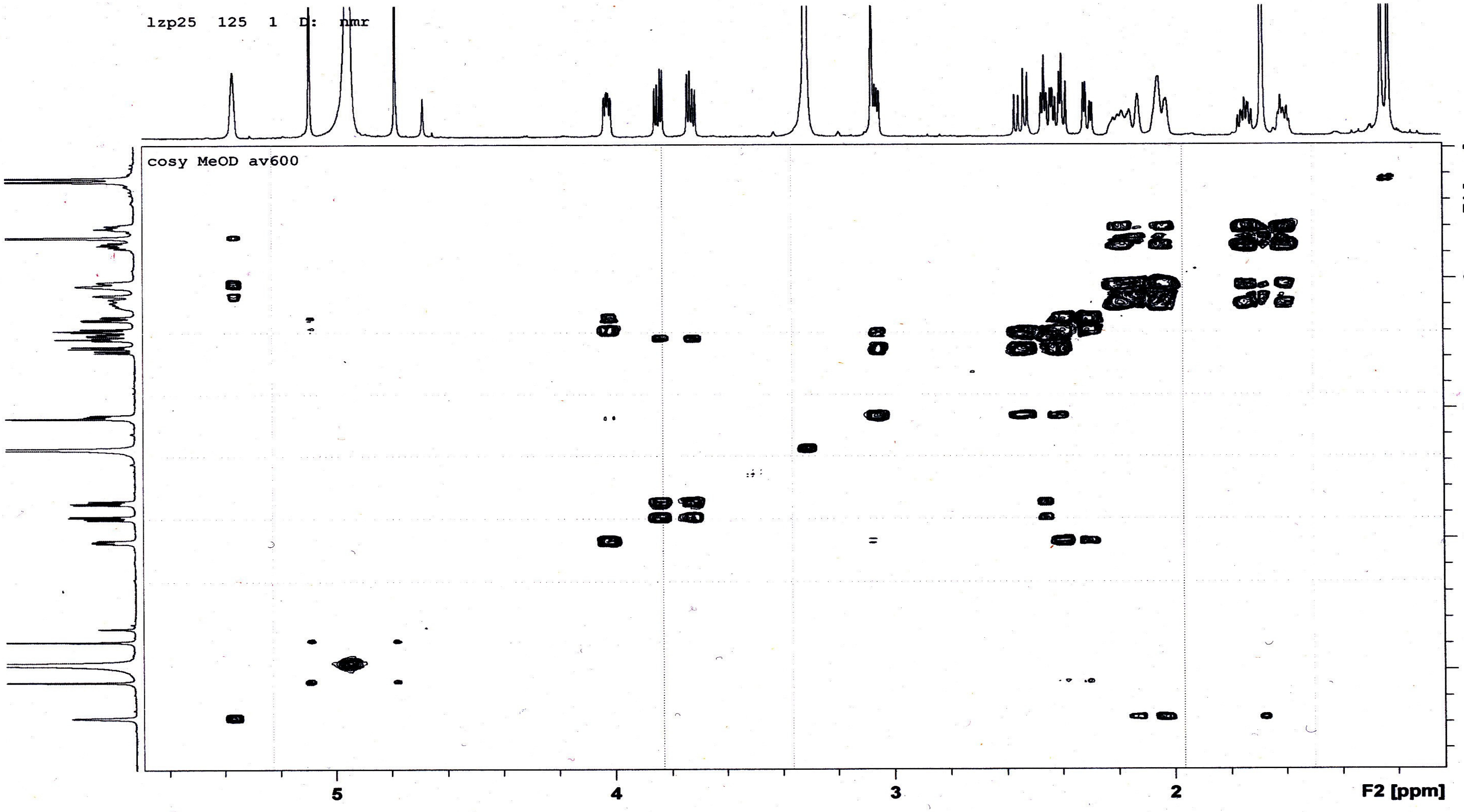

S6. ROESY spectrum (CD_3OD) of 8-oxoviscida-3,11(18)-diene-13,14,15,19-tetraol (**1**)

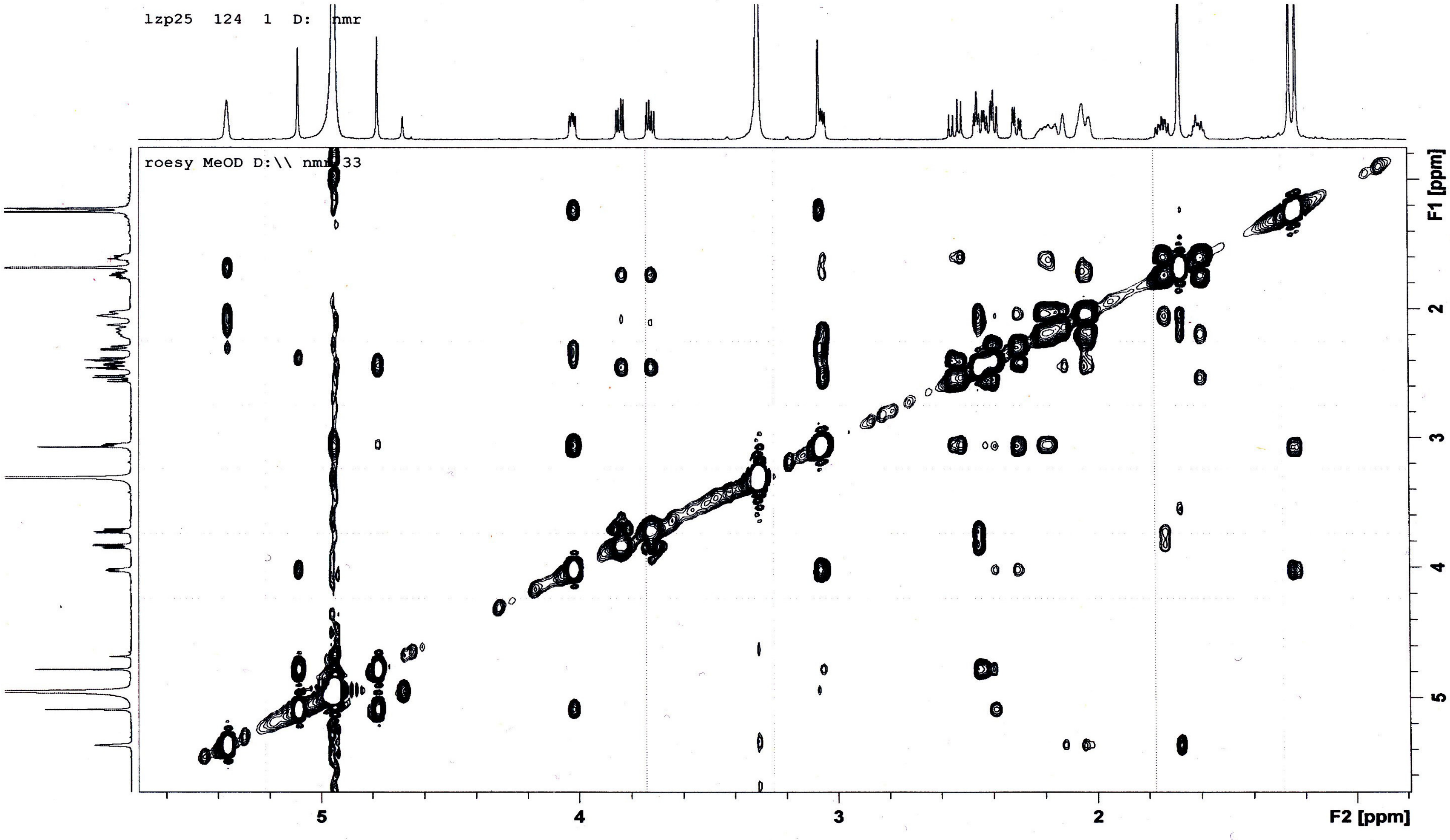

S7. ^{1}H NMR spectrum (CD_3OD) of (*E*)-10-(1,1-dimethyl-2-propenyloxy)-2-decene-4,6,8-triyn-1-ol (**2**)

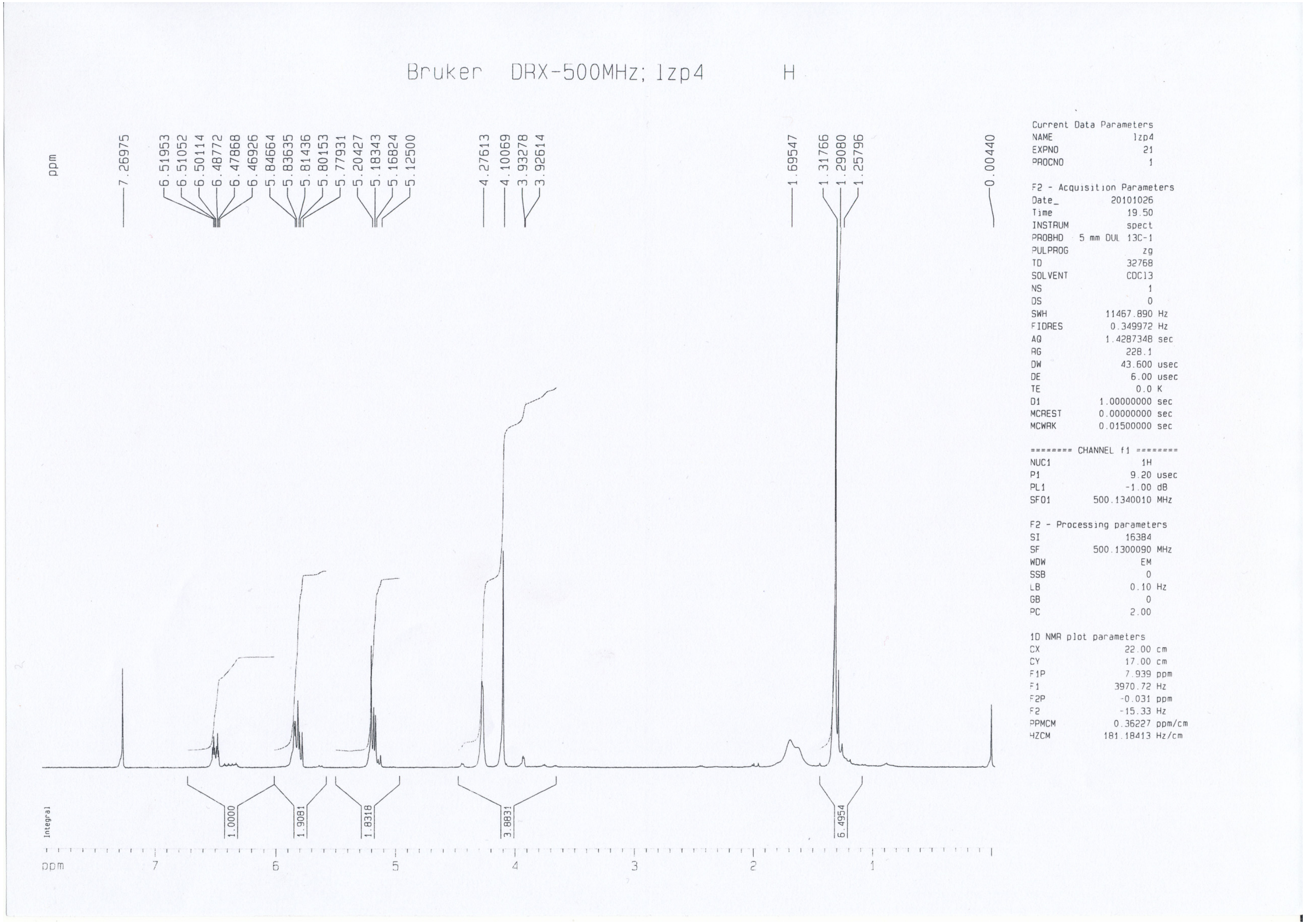

S8. ^{13}C NMR (DEPT) spectrum ($CDCl_3$) of (*E*)-10-(1,1-dimethyl-2-propenyloxy)-2-decene-4,6,8-triyn-1-ol (**2**)

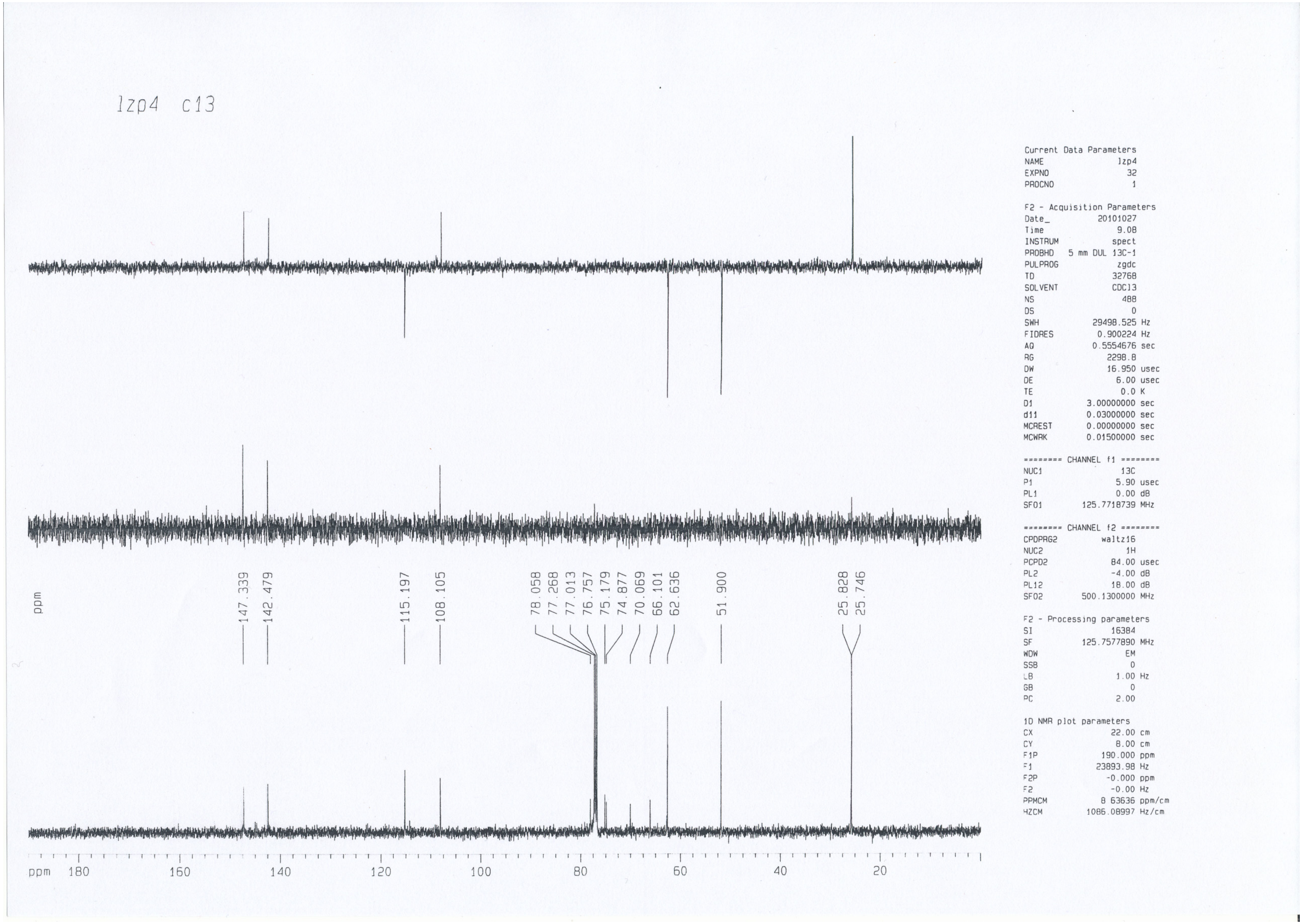

S9. HMBC spectrum ($CDCl_3$) of (*E*)-10-(1,1-dimethyl-2-propenyloxy)-2-decene-4,6,8-triyn-1-ol (**2**)

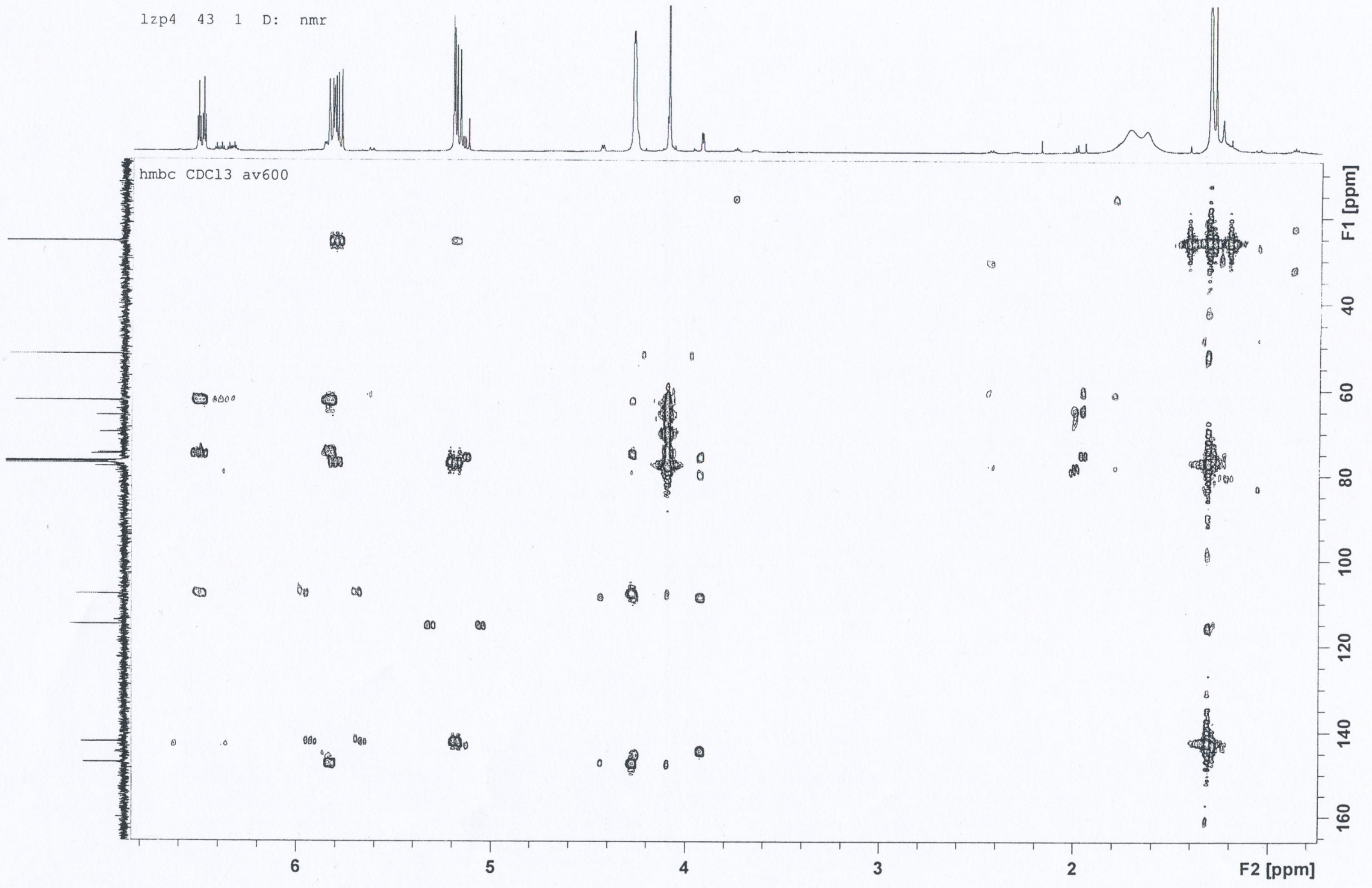

S10. HSQC spectrum ($CDCl_3$) of (*E*)-10-(1,1-dimethyl-2-propenyloxy)-2-decene-4,6,8-triyn-1-ol (**2**)

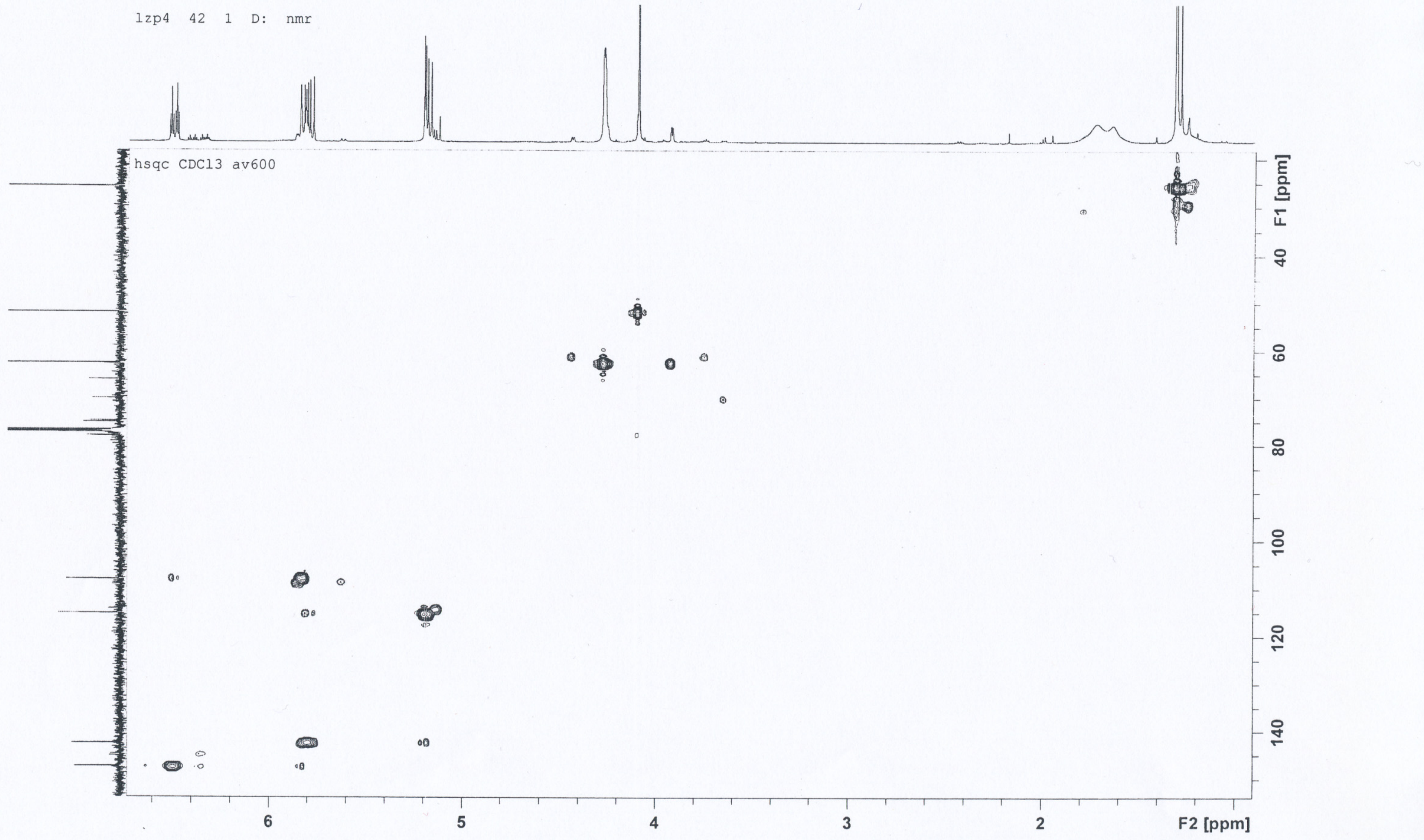

S11. ^{1}H NMR spectrum ($CDCl_3$) of 10-(1,1-dimethyl-2-propenyloxy)deca-4,6,8-triyn-1-ol (**3**).

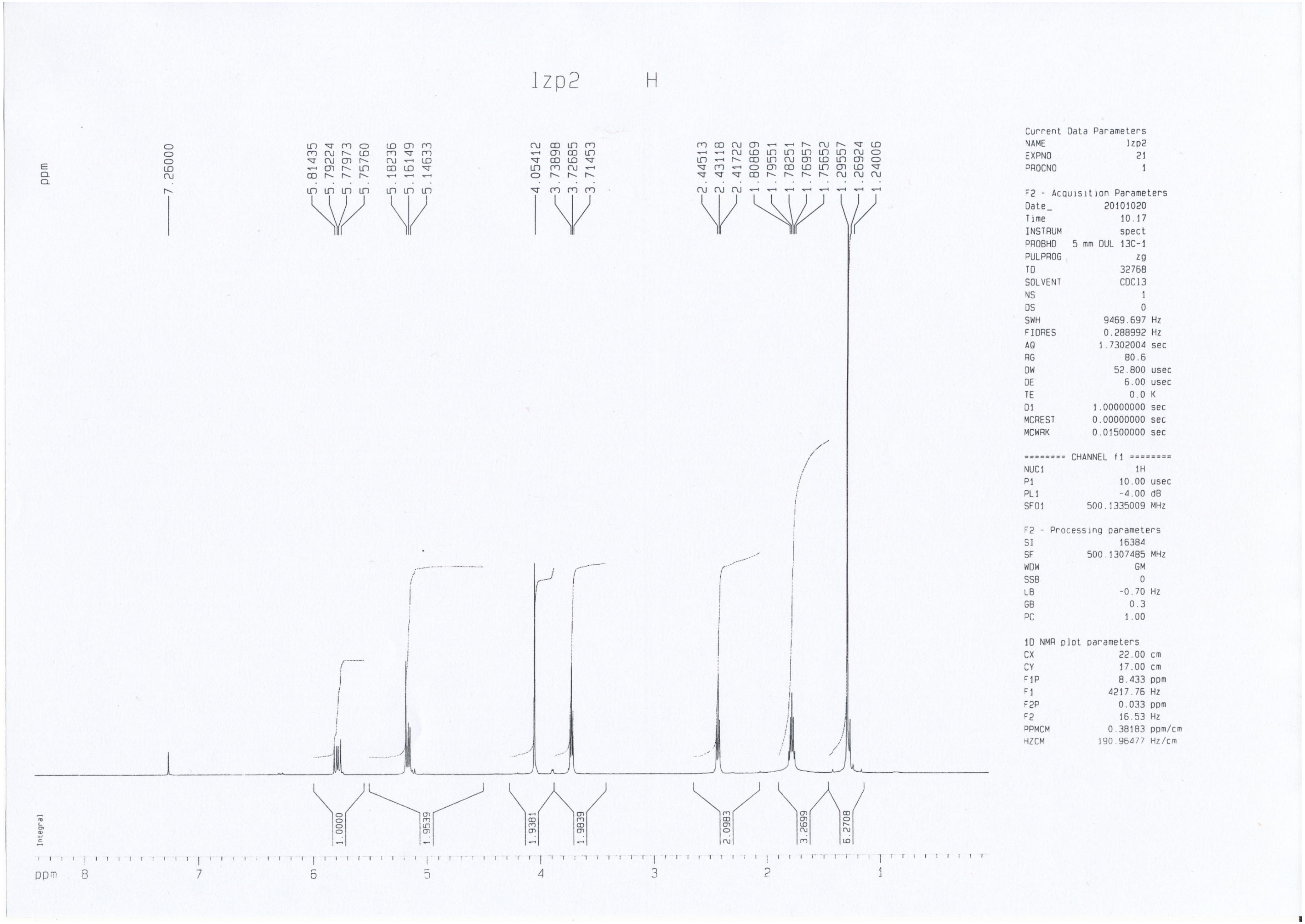

S12. ^{13}C NMR (DEPT) spectrum ($CDCl_3$) of 10-(1,1-dimethyl-2-propenyloxy)deca-4,6,8-triyn-1-ol (**3**)

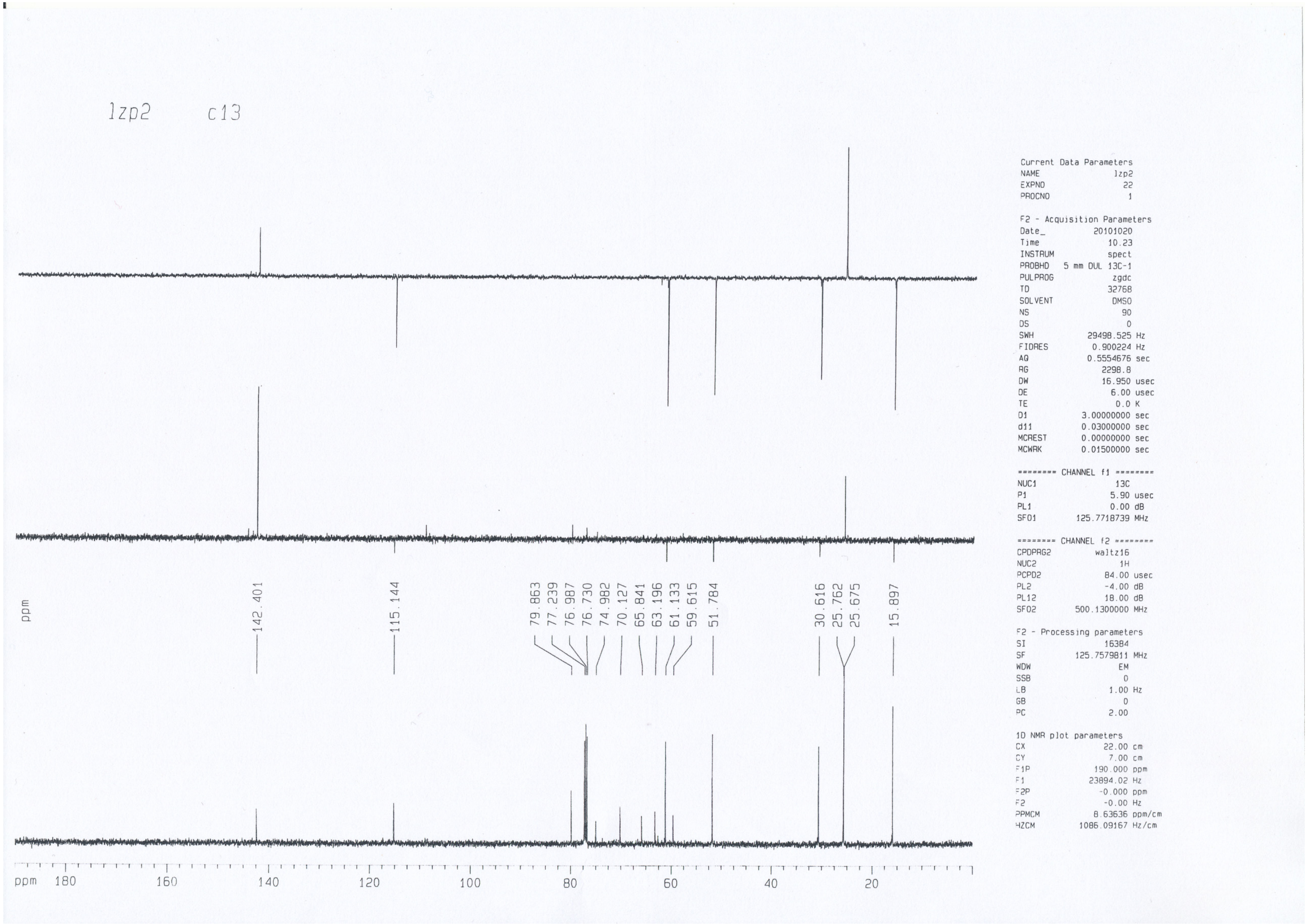

S13. HMBC spectrum ($CDCl_3$) of 10-(1,1-dimethyl-2-propenyloxy)deca-4,6,8-triyn-1-ol (**3**)

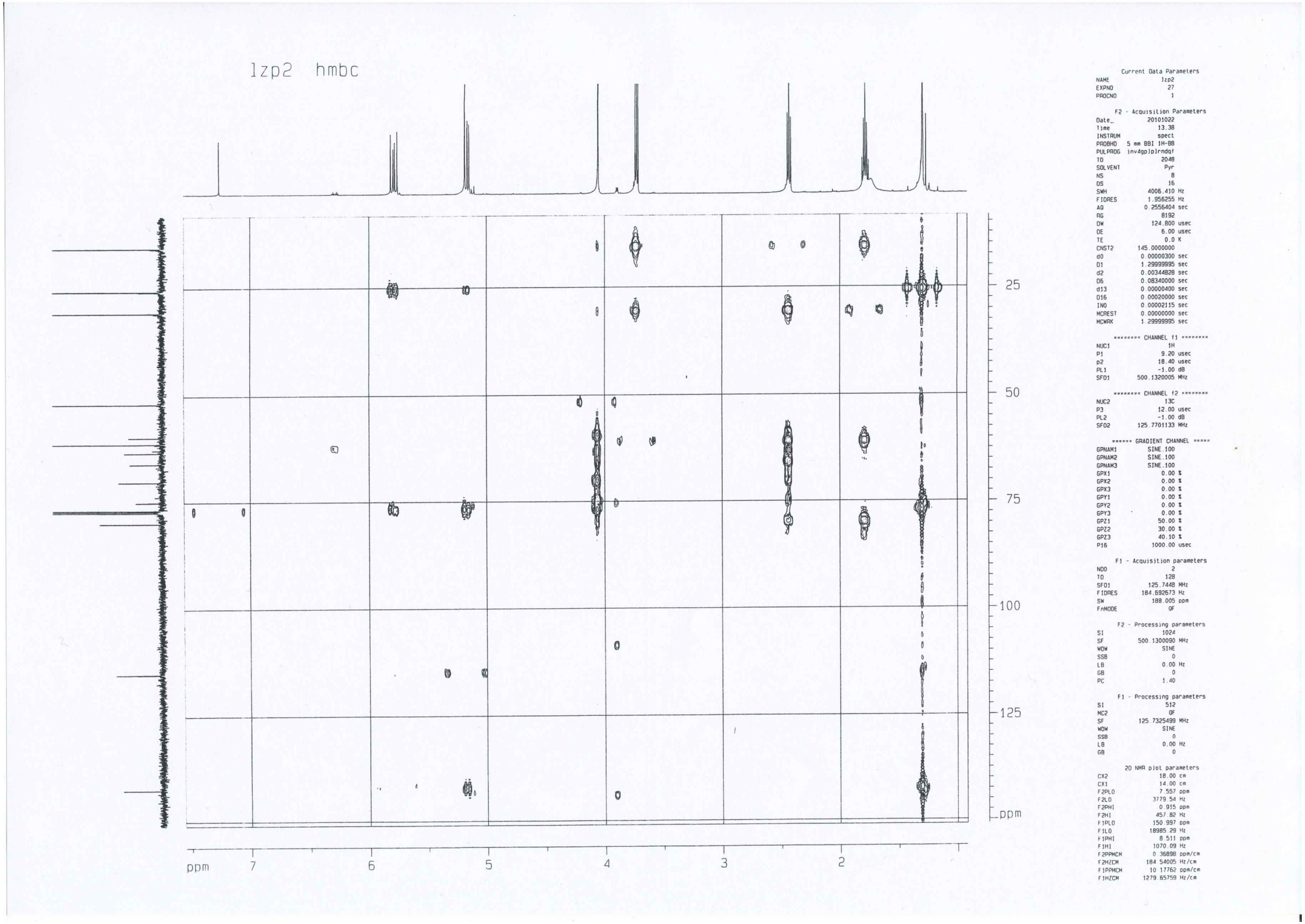

S14. HSQC spectrum ($CDCl_3$) of 10-(1,1-dimethyl-2-propenyloxy)deca-4,6,8-triyn-1-ol (**3**)

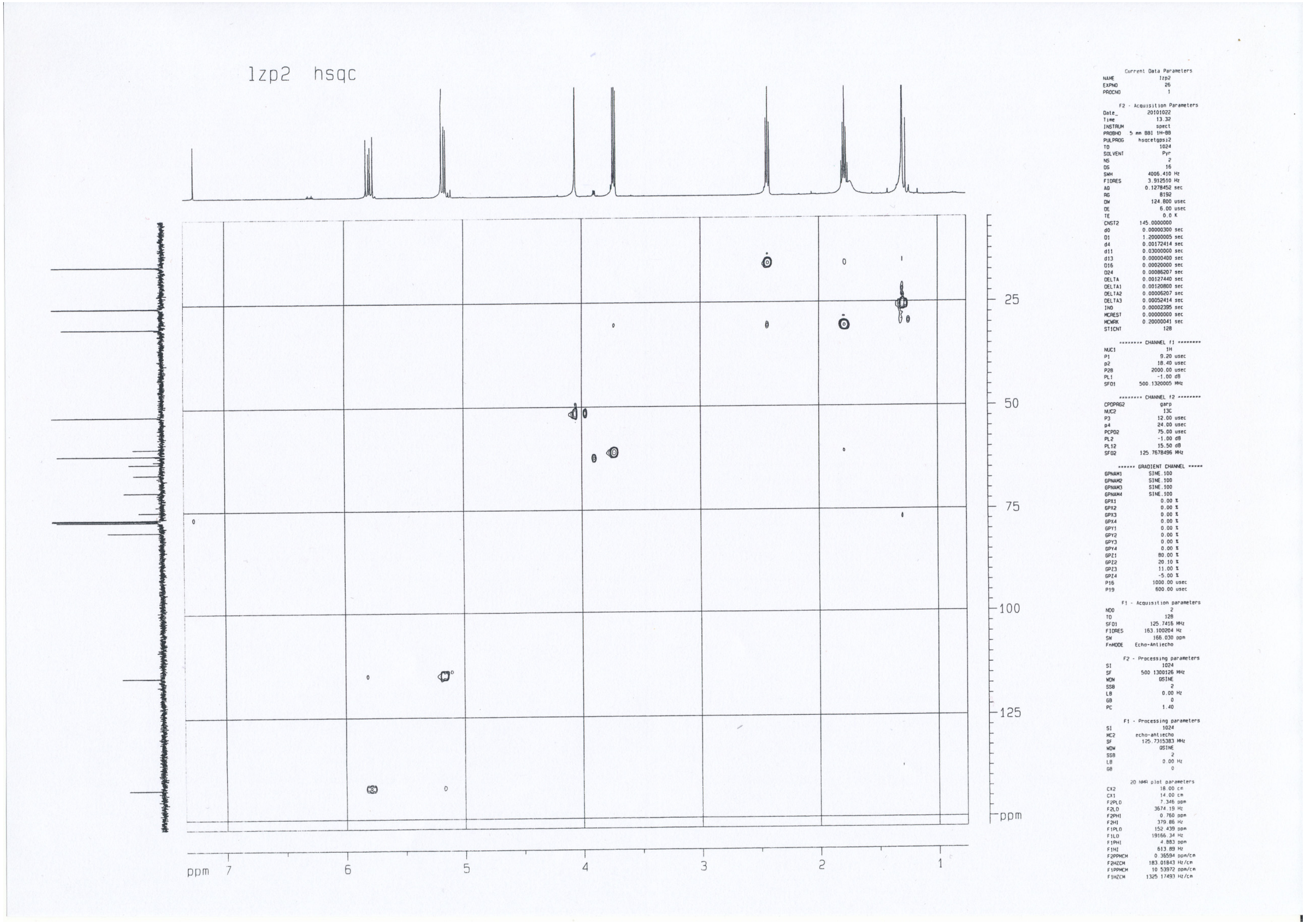

Supplement: Supplementary file 1 — Supplementary material 1 (PDF 10751 kb) [file 13659_2015_58_MOESM1_ESM.pdf]
